# Psychological resilience and emergency coping behavior among university freshmen: the mediating role of social support

**DOI:** 10.3389/fpubh.2026.1831841

**Published:** 2026-05-13

**Authors:** Yuezhu Wang

**Affiliations:** School of Civil Engineering, Anhui Jianzhu University, Hefei, China

**Keywords:** emergency coping behavior, psychological resilience, social support, structural equation modeling, university freshmen

## Abstract

**Introduction:**

University freshmen undergo a critical transition marked by separation from family, reconstruction of social networks, and adaptation to a new academic environment. Under these conditions, their capacity to respond effectively to emergencies may depend not only on internal psychological resources but also on access to external support resources. This study therefore examines the relationship between psychological resilience (PR) and emergency coping behavior (ECB) among university freshmen, with particular attention to the mediating role of social support (SS).

**Methods:**

Drawing on resilience theory, functional perspective on social support, and the transactional model of stress and coping, this study developed an integrated model linking PR, SS, and ECB. Questionnaire data were collected from 408 university freshmen, and structural equation modeling was used to test the proposed relationships.

**Results:**

PR was significantly associated with ECB, with tenacity, strength, and optimism related to more positive coping behavior and less negative coping behavior. PR was also significantly associated with emotional support and instrumental support, which were in turn associated with more positive coping behavior and less negative coping behavior. In addition, the SEM results were consistent with emotional support and instrumental support functioning as indirect pathways linking PR and ECB.

**Discussion:**

The findings suggest that ECB among university freshmen is meaningfully associated with both PR and SS. By identifying a correlational pattern consistent with an indirect pathway through SS, this study advances current understanding of how PR, SS, and ECB may be interrelated in emergency contexts. The results further underscore the importance of emotional and instrumental support as key domains for institutional attention during the transition to university life.

## 1 Introduction

University campuses constitute relatively concentrated social environments in which students engage in learning, living, and social interaction within shared spatial and organizational settings. Recent campus sustainability research further suggests that universities should be understood not only as educational organizations, but also as socio-ecological built environments in which campus design, microclimatic conditions, and sustainability-oriented infrastructure shape students’ health, comfort, and everyday functioning (1–3). Under such conditions, emergencies, defined here as sudden and disruptive event occurring in the campus context, may rapidly threaten students’ physical and mental health while also disrupting the continuity of academic and daily activities (4). Students’ ability to cope effectively with emergencies, such as fires, public health incidents, laboratory accidents, and acute health problems, is therefore particularly important, as it directly affects whether they can maintain emotional stability, make appropriate risk appraisals, mobilize available resources, and adopt timely and adaptive responses when emergencies occur (5). This issue is especially salient for university freshmen, who have just entered a new academic and social environment and are still adapting to unfamiliar routines, reconstructing interpersonal networks, and developing new support systems (6). As a result, when emergencies occur, university freshmen may be more vulnerable than senior students.

In such settings, how university freshmen perceive, regulate, and behaviorally respond to emergency-related demands becomes a critical issue, making emergency coping behavior (ECB) an important perspective for understanding student adaptation under stress. At the same time, ECB does not arise in isolation, but is closely related to the internal psychological resources and external support resources available to students when they encounter stress or emergencies. Among the internal resources that shape adaptation under stress, psychological resilience (PR) has been widely identified as a central protective factor. PR generally refers to the capacity to maintain, recover, or even strengthen psychological functioning in the face of adversity. A substantial body of research has shown that resilient individuals tend to display better emotional regulation, stronger stress tolerance, and more adaptive coping patterns (7). In student populations, higher resilience has consistently been associated with lower psychological distress and more constructive behavioral responses to challenge. However, the explanatory power of resilience remains incomplete if it is treated solely as an intrapersonal trait that directly determines coping outcomes. Emergency coping does not occur in a social vacuum. Particularly for freshmen, whether PR can be translated into effective action may depend not only on their internal psychological capacity, but also on the availability of support that can provide emotional reassurance, practical assistance, and guidance in unfamiliar settings.

This limitation points to the importance of social support (SS) as a key external resource linking psychological strengths to coping behavior. SS generally refers to the emotional, informational, and practical assistance that individuals perceive as available or actually receive through their relationships with family members, peers, teachers, and other significant others. In emergency situations, such support is particularly important because it can reduce uncertainty, alleviate emotional distress, and facilitate more timely and organized responses. For university freshmen, the role of SS is especially salient, as the transition to university often weakens previously familiar support systems while requiring the development of new ones. Under these conditions, students with stronger psychological resilience may be better able to perceive, seek, and utilize available support, and such support may in turn help translate internal psychological resources into more adaptive coping tendencies in emergency contexts (8).

Although previous studies have separately documented the positive role of PR in adaptation and the beneficial effects of SS on student well-being, three important gaps remain. First, existing research has largely examined PR, SS, and ECB in isolation, rather than integrating them into a unified explanatory framework. As a result, insufficient attention has been paid to how internal psychological resources are converted into adaptive coping through external support resources. Second, current research on university students has focused predominantly on general adjustment, mental health, or academic stress, while the specific formation mechanism of ECB among freshmen remains unexplored (8–10). This is a meaningful omission, because freshmen occupy a distinctive developmental and social position in which both personal regulation and network-based support are still unstable. Third, prior analyses often remain at the aggregate level, treating PR, SS, and ECB as broad constructs and thereby obscuring potentially important differences across their constituent dimensions. Yet the pathways connecting tenacity, strength, and optimism to positive and negative coping behavior may not be identical, nor are emotional support and instrumental support likely to operate in exactly the same way. Without a more differentiated analysis, the micro-level mechanism underlying freshmen’s ECB cannot be adequately understood.

To address these gaps, the present study examines the relationship between PR and ECB among university freshmen by integrating resilience theory, functional perspective on social support, and the transactional model of stress and coping. Specifically, this study proposes that PR may be associated with coping tendencies both directly and indirectly through the perception, access, and use of SS. This research contributes to the literature in three ways. First, it develops an integrated framework that links internal psychological resources, external support resources, and coping behavior within a single analytical model. Second, it identifies SS as a mediating mechanism, thereby clarifying how resilience may be transformed from a latent psychological capacity into concrete behavioral responses under stress. Third, by examining the multidimensional structure of PR, SS, and ECB, it provides a more fine-grained explanation of how freshmen respond to emergencies during the transition to university life. In doing so, the study not only enriches theoretical understanding of student adaptation and coping, but also offers practical implications for resilience cultivation, support network development, and emergency management in higher education.

## 2 Literature review and hypothesis development

### 2.1 Theoretical background

The transactional model of stress and coping conceptualizes coping under stress as a dynamic process arising from the interaction between situational demands and the internal and external resources available to the individual (11). In recent years, this model has been widely applied to examine the interrelationships among PR, self-efficacy, emotional states, SS, coping behavior, and mental health across specific populations and contexts, thereby providing an important theoretical basis for research on occupational health (12–14), the physical and psychological well-being of vulnerable groups (15–18), and behavioral responses under stressful conditions (19).

Relevant studies have shown that responses to stress depend largely on the internal and external resources available to people (20). Internal resources, such as PR, self-efficacy, and thinking style, enable individuals to maintain emotional stability, sustain confidence, and remain behaviorally engaged under pressure. External resources, mainly SS, provide emotional reassurance, informational guidance, and practical assistance that help individuals manage situational demands. Together, these internal and external resources are closely related to the direction and quality of coping behavior. When internal resources are stronger and external resources are more accessible, individuals are more likely to adopt positive coping behavior, such as problem solving, help seeking, and emotional regulation (21). By contrast, when these resources are insufficient, they are more likely to rely on negative coping behavior, such as avoidance, denial, withdrawal, or disorganized responses (22). Therefore, stress provides the situational basis for coping behavior, whereas internal and external resources function as key resources influencing the direction and quality of coping behavior.

This model is highly suitable for illustrating the ECB of university freshmen. When confronted with emergencies, freshmen often experience multiple sources of stress arising from unfamiliar environments, limited prior experience, and still-developing support networks. These stressors are commonly reflected in uncertainty and perceived threat, concern about potential consequences, anxiety regarding inadequate coping capacity, and the cognitive burden of making judgments and taking action under conditions of incomplete information. Under such circumstances, freshmen may adopt two broad patterns of response. One is positive coping behavior, including actively seeking authoritative information, turning to teachers or peers for assistance, mobilizing family and institutional support, and employing problem-solving and emotional regulation strategies. The other is negative coping behavior, including avoidance, excessive reliance on others, disorganized reactions under information overload, emotional venting, denial, or delay. Which pattern is ultimately adopted depends largely on whether freshmen possess sufficient internal psychological resources, have access to external support, and are able to maintain basic emotional stability and behavioral control under stress (23).

Among the internal resources relevant to ECB, PR has been widely recognized as a central protective factor. Resilience theory conceptualizes resilience as the capacity to maintain, recover, or achieve positive adaptation under adversity, and PR can therefore be understood as the individual-level psychological expression of this broader theoretical perspective. PR is generally defined as positive adaptation or effective functioning despite significant adversity (24). Within the transactional model of stress and coping, PR serves as a key internal resource that helps individuals remain emotionally stable, sustain confidence, and maintain behavioral engagement under pressure. Empirical research in university settings has consistently shown that PR influences coping behavior by enhancing individuals’ capacity to remain emotionally stable, sustain confidence, and maintain purposeful action when confronted with stress or emergencies (17). Individuals with higher resilience are generally better able to regulate negative emotions, tolerate uncertainty, and interpret stressful situations as manageable rather than overwhelming, which increases the likelihood of adopting adaptive coping strategies such as problem solving, help seeking, and emotional regulation (25). At the same time, PR can reduce the tendency toward maladaptive responses, including avoidance, denial, withdrawal, and disorganized reactions under pressure. In this sense, resilience may be associated not only with lower psychological strain under stress, but also with more adaptive coping tendencies through individuals’ stronger capacity to mobilize available resources and respond to situational demands in a constructive manner (26). Importantly, the function of PR is not limited to direct effects on ECB. Studies of first-year students have also shown that PR is positively associated with SS, suggesting that more resilient freshmen may be better able to perceive, seek, and mobilize supportive resources in unfamiliar environments (8). Taken together, these findings suggest that PR can be understood as a core internal resource that is associated with more positive coping behavior and greater access to external support.

Among the external resources relevant to emergency coping, SS has been widely recognized as a major contextual factor. SS generally refers to the emotional, informational, and practical assistance that individuals perceive as available or actually receive through their relationships with family members, peers, teachers, and other significant others. SS was initially defined as information that leads individuals to believe that they are cared for and loved, esteemed and valued, and part of a network of mutual obligation (27). In contemporary research, SS is more commonly understood as the emotional, informational, and instrumental assistance that individuals perceive as available or actually receive through their interpersonal relationships. For university freshmen, the role of SS is especially salient, as the transition to university often weakens previously familiar support systems while requiring the development of new ones. Available SS of them mainly includes emotional reassurance, informational guidance, practical assistance, and institutional support from family members, peers, teachers, counselors, and university organizations. These SS can not only reduce the psychological strain experienced by university freshmen during emergencies, but also enhance their likelihood of seeking help, mobilizing resources, and adopting positive coping behavior, while reducing maladaptive tendencies such as avoidance, delay, and disorganization (28–30). Taken together, these findings suggest that SS can be understood as a core external resource that is closely related to coping behavior and may also represent an important indirect pathway linking internal psychological resources to coping outcomes.

Overall, the transactional model of stress and coping provides an overarching framework for understanding ECB among university freshmen by highlighting the joint role of situational stress and available resources. Within this framework, PR functions as a core internal resource that supports emotional stability, adaptive action, and the mobilization of external support, whereas SS represents a key external resource that provides emotional reassurance, informational guidance, and practical assistance. Accordingly, ECB among university freshmen may be understood as a correlational outcome of the interplay between PR and SS, with SS representing an important pathway through which PR may be linked to positive coping behavior. This integrated perspective provides the theoretical basis for the hypotheses proposed in the following section.

### 2.2 Research hypothesis

#### 2.2.1 Psychological resilience and emergency coping behavior

In emergency situations, university freshmen often face uncertainty, limited experience, and unstable self-regulation. In the present study, PR was divided into three dimensions—tenacity, strength, and optimism—primarily based on the factor structure of the Chinese version of the Connor–Davidson Resilience Scale (CD-RISC). The original CD-RISC developed by Connor and Davidson was a 25-item instrument and was initially reported as a five-factor measure (31). However, subsequent studies have shown that the original five-factor structure is not consistently replicated across different cultural contexts and samples. In particular, Yu and Zhang conducted a psychometric evaluation of the Chinese version of the CD-RISC and found that the original five-factor model did not fit the Chinese data well, whereas exploratory factor analysis supported a three-factor structure labeled tenacity, strength, and optimism (32). Since then, this three-dimensional structure has been widely adopted in Chinese research contexts and is considered more appropriate for capturing the construct of PR among Chinese participants. Therefore, the present study followed this culturally validated classification and operationalized PR in terms of tenacity, strength, and optimism.

Tenacity generally refers to persistence and psychological stability under pressure. Existing studies on resilience and coping suggest that individuals with higher persistence are more likely to remain engaged in stressful situations and less likely to disengage when facing disruption or difficulty, although direct evidence in emergency-specific contexts remains limited. In this sense, tenacity may help university freshmen sustain problem-focused efforts and reduce withdrawal or avoidance when emergencies occur. Strength reflects a positive evaluation of one’s own capacity to deal with adversity. Prior research has consistently indicated that stronger self-belief is associated with greater perceived controllability and a higher likelihood of adopting active coping responses, such as information seeking, support mobilization, and purposeful action. By contrast, lower levels of perceived personal strength have more often been linked to helplessness and passive responding. Optimism refers to positive expectations regarding future outcomes. Empirical evidence generally suggests that optimistic individuals are less likely to interpret stressful situations as overwhelming and may be better able to regulate negative emotions, which in turn is associated with more constructive coping and lower reliance on avoidant responses. However, the magnitude and consistency of these effects may vary across contexts and populations (32).

Taken together, the existing literature suggests that PR may influence ECB through several interrelated pathways, including emotional stability, perceived coping capacity, and expectations about outcomes. Although prior studies have rarely examined these relationships specifically in the context of university freshmen’s emergency coping, the available evidence provides a reasonable basis for expecting that higher levels of resilience will be associated with more positive coping behavior and less negative coping behavior. Accordingly, the following hypotheses are proposed:

*H*1: Psychological resilience significantly influences emergency coping behavior among university freshmen.

*H*1a: Tenacity is positively associated with positive coping behavior and negatively associated with negative coping behavior.

*H*1b: Strength is positively associated with positive coping behavior and negatively associated with negative coping behavior.

*H*1c: Optimism is positively associated with positive coping behavior and negatively associated with negative coping behavior.

#### 2.2.2 Psychological resilience and social support

SS represents an important external resource through which individuals receive or perceive assistance from significant others when facing stress or difficulty. Related research has consistently shown that SS can be broadly differentiated into two core forms: emotional support, which provides empathy, reassurance, trust, and a sense of psychological security, and instrumental support, which provides tangible help, practical assistance, and active coping aid (33). This distinction is especially appropriate in the context of university freshmen facing emergencies, because the support they can access is typically manifested either as emotional reassurance that reduces anxiety and stabilizes emotion, or as practical help and guidance that facilitates action and problem solving. Empirical studies in college populations further indicate that different profiles of perceived support are associated with different coping tendencies and resilience-related functioning, while individual-level SS can operate as an important mechanism linking crisis exposure to psychological outcomes (34). In such a context, the development of emotional and instrumental support depends in part on whether students can engage with others in a stable, open, and adaptive way. PR may therefore foster SS by shaping how freshmen form, sustain, and mobilize social relationships.

At the dimensional level, this relationship may be further specified, although the available evidence remains more suggestive than conclusive. Tenacity generally reflects persistence and psychological stability in the face of difficulty. Existing studies on resilience and social support suggest that individuals with greater persistence are more likely to remain engaged in interpersonal relationships despite uncertainty, misunderstanding, or initial setbacks. For university freshmen, such relational persistence may increase the likelihood of maintaining interaction over time and, consequently, may facilitate the development of both emotional and instrumental support. Strength refers to a positive judgment of one’s own capacity to cope with adversity. Prior research has indicated that stronger perceived competence is often associated with greater willingness to express needs, seek assistance, and approach others with confidence. In the context of university transition, this tendency may be especially relevant to instrumental support, because access to information, advice, and practical help frequently depends on proactive help-seeking. Thus, strength may be more strongly related to instrumental support, while also contributing to supportive relationships more broadly. Optimism reflects positive expectations regarding future outcomes and, by extension, social interaction. Some studies have suggested that optimistic individuals are generally less defensive and more likely to trust others, which may facilitate the formation of affective bonds (35). Among university freshmen, this tendency may be particularly relevant to emotional support, as it may encourage reciprocity, interpersonal closeness, and a stronger sense of belonging. At the same time, positive expectations may also contribute to broader supportive networks.

Taken together, the existing literature suggests that PR may contribute to SS by strengthening relational persistence, increasing confidence in help-seeking, and fostering more positive interpersonal expectations. On this basis, it is reasonable to expect that higher levels of resilience will be associated with greater emotional support and instrumental support among university freshmen. Accordingly, the following hypotheses are proposed:

*H*2: Psychological resilience is positively associated with social support among university freshmen.

*H*2a: Tenacity is positively associated with both emotional support and instrumental support.

*H*2b: Strength is positively associated with both emotional support and instrumental support, with a stronger association with instrumental support.

*H*2c: Optimism is positively associated with both emotional support and instrumental support, with a stronger association with emotional support.

#### 2.2.3 Social support and emergency coping behavior

SS may shape ECB by influencing both emotional regulation and response capacity. For university freshmen, who often confront emergencies with limited experience and still-developing support networks, the availability of support resources can substantially affect how they interpret stressful events and how they respond to them (36). In the present study, ECB was divided into positive coping behavior and negative coping behavior primarily on both theoretical and measurement grounds. Theoretically, coping research rooted in the stress and coping tradition has long suggested that individuals’ responses to stress can be broadly distinguished into more adaptive, approach-oriented efforts and more maladaptive, avoidant or disengaged responses, indicating that coping is not unidimensional but differs in direction and function (11, 37). Empirically, this distinction is consistent with the Simplified Coping Style Questionnaire (SCSQ), which was developed by Xie on the basis of the Ways of Coping Questionnaire and adapted to the Chinese context (38). The SCSQ explicitly classifies coping into two dimensions, namely positive coping behavior and negative coping behavior, and this two-factor structure has been widely used and validated in Chinese student samples. Positive coping behavior mainly reflects active and constructive responses such as problem solving, help seeking, and positive reinterpretation, whereas negative coping behavior reflects avoidant or maladaptive responses such as withdrawal, denial, and reliance on passive solutions.

Emotional support may influence coping by reducing distress and strengthening psychological security. Existing research has generally suggested that individuals who perceive greater emotional support tend to regulate anxiety more effectively, maintain greater emotional stability, and show less vulnerability to panic-driven reactions in stressful situations (39). For university freshmen, who are still adapting to a new environment and support system, this buffering function may be especially relevant. In such cases, emotional support may increase the likelihood of more adaptive responses, such as rational problem solving and appropriate help-seeking, while reducing the tendency toward maladaptive responses, including avoidance, denial, or emotional disengagement (40).

Instrumental support appears to operate through a more functional pathway. Prior studies have indicated that access to timely information, concrete advice, and practical assistance can improve situational understanding and facilitate more effective action under stress. This may be particularly important for university freshmen, who often have limited experience in assessing risks and determining appropriate responses independently. When such support is available, they may be better positioned to interpret the situation, make appropriate decisions, and implement more effective coping responses. In this sense, instrumental support may be associated with greater positive coping behavior and lower levels of negative coping behavior arising from uncertainty, confusion, or lack of guidance (36).

Taken together, the existing literature suggests that emotional support and instrumental support may contribute to emergency coping in complementary ways. Emotional support appears more relevant to psychological stabilization under stress, whereas instrumental support may be more closely related to response capacity and effective action. Although prior evidence has not always been derived specifically from university freshmen in emergency contexts, it provides a reasonable basis for expecting that both forms of support will be positively associated with positive coping behavior and negatively associated with negative coping behavior among university freshmen. Accordingly, the following hypotheses are proposed:

*H*3: Social support significantly influences emergency coping behavior among university freshmen.

*H*3a: Emotional support is positively associated with positive coping behavior and negatively associated with negative coping behavior.

*H*3b: Instrumental support is positively associated with positive coping behavior and negatively associated with negative coping behavior.

#### 2.2.4 The mediating role of social support

PR may influence ECB not only directly, but also indirectly through SS. For university freshmen, the adaptive value of resilience may depend in part on whether internal psychological resources can be translated into accessible interpersonal support. In this sense, SS may function as an important mechanism linking PR to ECB, although existing evidence remains more indicative than definitive, particularly in emergency-specific contexts involving freshmen (41).

One possible indirect pathway may operate through emotional support. Prior research has generally suggested that individuals with higher levels of resilience are more likely to maintain trust, openness, and relational stability in unfamiliar or stressful social settings. Among university freshmen, these tendencies may facilitate the formation of affective ties and increase the likelihood of perceiving emotional support as available when emergencies arise. Existing studies also indicate that emotional support can alleviate anxiety, strengthen psychological security, and help individuals remain emotionally regulated under stress. Accordingly, it is reasonable to expect that the influence of PR on ECB may be partly transmitted through emotional support, thereby promoting positive coping behavior and reducing negative coping behavior.

A similar process may occur through instrumental support. Freshmen with stronger PR, particularly those with greater perceived personal strength, may be more willing to express needs clearly, seek help proactively, and mobilize external assistance when facing uncertainty. Such tendencies may increase their access to instrumental support, including useful information, concrete guidance, and practical help. Previous research has suggested that these resources can improve situational understanding, facilitate more effective decision-making, and strengthen response capacity under stress. Therefore, PR may also be indirectly associated with ECB through instrumental support, contributing to greater positive coping behavior and lower levels of negative coping behavior.

Taken together, this mediating relationship can be viewed as a theoretically grounded expectation that SS may serve as a mediating mechanism through which PR influences ECB among university freshmen. On this basis, the following hypotheses are proposed:

*H*4: Social support mediates the relationship between psychological resilience and emergency coping behavior among university freshmen.

*H*4a: Emotional support mediates the relationship between the dimensions of psychological resilience and both positive coping behavior and negative coping behavior.

*H*4b: Instrumental support mediates the relationship between the dimensions of psychological resilience and both positive coping behavior and negative coping behavior.

#### 2.2.5 Conceptual model development

**Figure 1:**
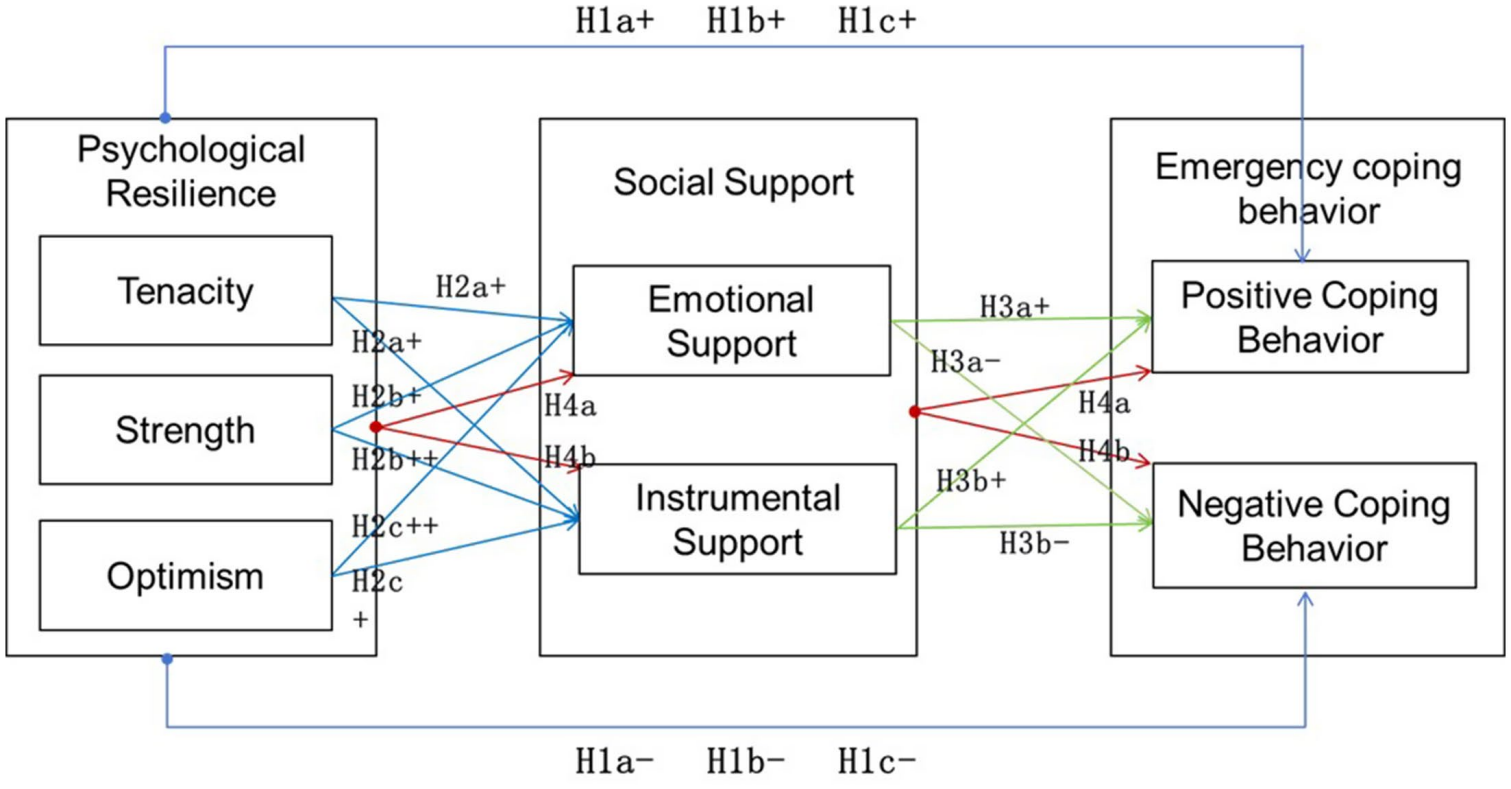
Conceptual model of the relationship between PR, SS, and ECB.

Drawing on resilience theory, functional perspective on social support, and the transactional model of stress and coping, this study develops a theoretical model of ECB among university freshmen, as shown in Figure 1. In this model, PR is conceptualized as the antecedent variable, SS as the mediating variable, and ECB as the outcome variable, with the aim of systematically explaining the underlying formation mechanism and pathways of ECB among university freshmen. Specifically, this study proposes that PR is associated with ECB not only through its direct relationship with coping tendencies, but also through the perceived availability and effective use of SS. This theoretical model provides the conceptual basis for the development of the research hypotheses and the subsequent empirical analysis.

### 2.3 Questionnaire survey

A structured self-report questionnaire was used to measure PR, SS, and ECB among university freshmen. Because all focal constructs in this study were assessed using established and validated instruments, no *de novo* questionnaire development procedure was undertaken; however, the instruments were organized and clarified in accordance with the conceptual model and survey context of the present study. The final questionnaire consisted of two sections. The first section collected demographic information, including gender, age, place of origin, only-child status, and academic discipline. The second section comprised 55 psychometric items, including 25 items for PR, 10 items for SS, and 20 items for ECB. Thus, the full questionnaire contained 60 items in total.

PR was measured using the 25-item Chinese version of the Connor–Davidson Resilience Scale (CD-RISC-25) psychometrically validated in Chinese samples by Yu and Zhang (32). Tenacity was measured by items 11–21, strength by items 1, 5, 7–10, 24, and 25, and optimism by items 2, 3, 4, and 6. Sample items included statements reflecting persistence in the face of difficulty, confidence in dealing with challenges, and a positive outlook toward future outcome. These items were used to capture intrapersonal adaptive capacity rather than practical support obtained from others.

SS was measured using the 10-item Social Support Rating Scale (SSRS) developed by Xiao, a widely used instrument in Chinese research for assessing individuals’ (42). In the present study, to align the measurement with the proposed theoretical model, SSRS items were interpreted according to their functional meaning and used to represent two support-based dimensions of SS: emotional support and instrumental support. Emotional support primarily reflected perceived care, reassurance, and relational closeness, as captured by items on the availability of close friends, the frequency of interaction with classmates or neighbors, support and care received from family members, and sources of comfort and concern in emergency situations. It was measured by items 1, 3–5. Instrumental support primarily reflected tangible and actionable assistance, as captured by items on living arrangements, sources of economic or practical help in emergencies, help-seeking behavior when facing trouble, and participation in organized group activities. It was measured by items 2, 6, and 7. Items 8–10 were used to assess support utilization, which reflects the extent to which individuals actively seek, access, and make use of available support when facing difficulties. In the present study, however, support utilization was not modeled as an independent dimension of SS. This is because the study focuses on the availability of external support resources rather than on students’ behavioral tendency to use such resources. More importantly, support utilization involves active help-seeking and problem-solving responses, which may overlap conceptually with emergency coping behavior. The SSRS was selected because it captures key forms of support available to university freshmen, particularly emotional reassurance and practical assistance, and is therefore consistent with the present study’s focus on support resources in emergency-related coping contexts.

ECB was measured using the 20-item Simplified Coping Style Questionnaire (SCSQ) developed by Xie on the basis of the Ways of Coping Questionnaire and adapted to the Chinese context (38). The SCSQ classifies coping into two dimensions: positive coping behavior and negative coping behavior. Positive coping behavior was measured by items 1 to 12, and negative coping behavior was measured by items 13 to 20. Positive coping behavior items mainly reflect active and constructive responses, such as seeking advice, trying to solve problems, and focusing on the manageable side of the situation, whereas negative coping behavior items mainly reflect avoidance-oriented or passive responses, such as waiting for the problem to disappear, attempting to forget it, or relying on ineffective distraction.

To reduce conceptual ambiguity among the three constructs, the present study distinguished them at both the theoretical and measurement levels. PR was operationalized as an internal adaptive resource, with items primarily assessing persistence, self-confidence, and optimism under adversity. SS was operationalized as an external supportive resource, with items focusing on the emotional reassurance, practical assistance, and accessible help that university freshmen may perceive or receive from family members, peers, teachers, counselors, and other relevant support providers. ECB was operationalized as a behavioral response tendency, with items describing how individuals typically act when facing stress or difficulty. Although the CD-RISC-25 includes one item that is indirectly related to knowing where to obtain help, the scale as a whole is intended to assess individuals’ perceived adaptive capacity, including persistence, confidence, and optimism under adversity. In contrast, the SSRS is specifically designed to assess the availability and receipt of SS from interpersonal and social sources, such as family members, peers, teachers, and other relevant support providers. The distinction between the constructs was further examined empirically in the measurement model, and the results supported acceptable discriminant validity.

Table 1 lists the partial measurement items of each dimension. All items were rated using the original scoring methods of the respective scales. The use of these established instruments helped ensure the reliability and validity of the measurements and provided a sound basis for the subsequent empirical analysis.

**Table 1:** Partial measurement items of each dimension.

Concepts	Dimension	Partial measurement item
Psychological resilience	Tenacity	I can achieve my goals.
		When things look hopeless, I do not give up easily.
	Strength	I am able to adapt to change.
		Past successes give me confidence in dealing with new challenges.
	Optimism	I have close and secure relationships.
		Sometimes fate or God can help.
Social support	Emotional support	How many close friends do you have who can provide you with support and help?
		Your relationship with your classmates or colleagues is?
	Instrumental support	In the past year, your living situation has mainly been?
		In times of emergency or difficulty, what sources have provided you with financial support or practical help?
Emergency coping behavior	Positive coping behavior	I try to get rid of my troubles through work or study.
		I talk with others and express my inner distress.
	Negative coping behavior	I relieve my troubles by smoking, drinking, taking medicine, or eating.
		I believe that time will change the situation, and the only thing to do is wait.

Before the formal survey, a small-scale pilot administration was conducted with a group of freshmen to check the clarity of instructions, item comprehension, and completion time. Based on this process, only minor adjustments were made to the wording of the questionnaire instructions and item ordering, while the substantive content of the original scales remained unchanged. The questionnaire survey was conducted among university freshmen. Before the formal survey, respondents were informed of the purpose of the study and assured that their responses would be used for academic research only and kept confidential. This study was reviewed and approved by the School of Civil Engineering, Anhui Jianzhu University (Ethics Approval No. CE-AHJZU-2025-008). The study involved only interviews and questionnaire surveys and did not include any drug administration, medical intervention, physiological sampling, invasive procedures, or other harmful experiments on human participants. All participants provided informed consent prior to participation, and all data were collected anonymously and used solely for academic research purposes.

A class-based convenience sampling strategy was adopted in the present study. The survey was conducted among full-time first-year undergraduate students at Anhui Jianzhu University. With the assistance of instructors and student counselors, questionnaires were distributed in classroom-based or student management settings during the regular academic term. Before the formal survey, all participants were informed of the purpose of the study, the voluntary nature of participation, the anonymity of responses, and the confidentiality of the data. Students who agreed to participate completed the questionnaire independently on site, and no financial or material incentive was provided. Only questionnaires completed by university freshmen were considered eligible for analysis. The returned questionnaires were screened according to the following criteria: respondents had to be first-year undergraduate students; questionnaires with substantial missing data were excluded; questionnaires showing obvious response regularity were removed; and questionnaires containing inconsistent or invalid demographic information were also excluded. A total of 600 questionnaires were distributed, of which 408 valid questionnaires were retained after data screening, yielding an effective response rate of 68.00%. The final sample size was considered adequate for the subsequent empirical analysis. The demographic characteristics of the valid sample are presented in Table 2. Among the respondents, most participants were aged 18 years, 64.71% of the respondents came from urban areas, and most of the respondents were only children.

**Table 2:** Demographic characteristics of surveyed university freshmen.

Measurement	Variable options	Number	Percentage (%)
Gender	Male	220	53.92
	Female	188	46.08
Age	17 years	65	15.93
	18 years	287	70.34
	19 years and above	56	13.73
Place of origin	Urban	264	64.71
	Rural	144	35.29
Only-child status	Yes	293	71.81
	No	115	28.19
Academic discipline	Science	70	17.16
	Engineering	112	27.45
	Medicine	58	14.22
	Economics	42	10.29
	Law	28	6.86
	Literature	36	8.82
	Philosophy	10	2.45
	Arts	24	5.88
	Others	28	6.86

To test the proposed hypotheses, statistical analyses were conducted using SPSS 26.0 and AMOS 31.0. First, descriptive statistics were performed to summarize the basic characteristics of the sample and examine the distribution of the data. Second, reliability analysis and confirmatory factor analysis (CFA) were conducted to assess the measurement properties of the scales, including internal consistency, convergent validity, and discriminant validity. Third, structural equation modeling (SEM) was employed to examine the direct effects of PR on SS and ECB, as well as the effects of SS on ECB. Finally, the mediating role of SS was tested using the bias-corrected bootstrap method. Given its ability to simultaneously estimate multiple relationships among latent variables and to test mediating mechanisms, SEM was considered appropriate for the present study.

## 3 Results

### 3.1 Data distribution check

As shown in Table 3, descriptive statistics confirmed the data’s adherence to a normal distribution, with skewness values ranging from −0.86 to 1.56 and kurtosis values between −2.00 and 0.43, all within acceptable thresholds. These results validate the suitability of the dataset for further statistical analysis.

**Table 3:** Descriptive statistical analysis of measurement item.

Item	Mean	Skewness	Kurtosis	Item	Mean	Skewness	Kurtosis	Item	Mean	Skewness	Kurtosis
A11	2.27	−0.34	−0.70	A24	2.62	−0.46	−0.19	C4	2.59	−0.20	−1.10
A12	2.51	−0.40	−0.90	A25	2.52	−0.47	−0.11	C5	2.62	−0.22	−1.01
A13	2.41	−0.40	−0.73	A2	2.39	−0.34	−0.44	C6	2.71	−0.31	−0.98
A14	2.25	−0.22	−0.93	A3	2.49	−0.52	−0.52	C7	2.57	−0.10	−1.04
A15	2.35	−0.25	−0.74	A4	2.29	−0.36	−0.89	C8	2.58	−0.11	−1.19
A16	2.46	−0.48	−0.76	A6	2.78	−0.86	−0.04	C9	2.67	−0.25	−1.03
A17	2.52	−0.32	−0.47	B1	2.41	0.05	−0.56	C10	2.71	−0.39	−0.98
A18	2.63	−0.45	−0.85	B3	2.63	−0.13	−1.19	C11	2.65	−0.27	−1.04
A19	2.23	−0.04	−1.06	B4	2.66	−0.16	−1.15	C12	2.73	−0.37	−1.02
A20	2.45	−0.36	−0.99	B5	11.05	−0.18	−1.23	C13	1.96	0.40	−0.84
A21	2.43	−0.27	−1.06	B2	2.30	0.25	−1.08	C14	2.00	0.22	−1.04
A22	2.19	−0.19	−1.14	B6	3.13	0.49	0.07	C15	1.95	0.41	−0.89
A23	2.48	−0.43	−1.02	B7	2.78	0.72	0.08	C16	2.00	0.28	−0.90
A1	2.48	−0.35	−0.32	B8	2.52	0.13	−1.10	C17	1.92	0.36	−1.06
A5	2.36	−0.40	−0.21	B9	2.37	0.24	−1.12	C18	1.92	0.52	−0.65
A7	2.49	−0.56	−0.32	B10	2.40	0.20	−1.17	C19	1.94	0.39	−0.92
A8	2.56	−0.23	−0.34	C1	2.51	−0.05	−1.29	C20	1.88	0.42	−1.00
A9	2.43	−0.67	−0.17	C2	2.58	−0.16	−1.16				
A10	2.61	−0.66	−0.58	C3	2.65	−0.24	−1.01				

### 3.2 Measurement model assessment

1. Reliability test.

To assess internal consistency, a reliability test was conducted, with Cronbach’s alpha values of 0.7 or higher considered acceptable. The analysis revealed Cronbach’s alpha values of 0.95 for Tenacity, 0.90 for Strength, 0.84 for Optimism, 0.92 for EmS, 0.87 for InS, 0.95 for PCB, and 0.90 for NCB, indicating strong internal consistency across all constructs. Corrected item-total correlations (CITC) for all items ranged from 0.65 to 0.83, exceeding the recommended threshold of 0.3 and affirming their relevance to their respective constructs. Additionally, Cronbach’s alpha values remained lower after item deletion, confirming that all items contributed positively to the scales’ reliability. Collectively, these results validate the reliability of the measurement model.

1. Confirmatory factor analysis and validity test.

**Figure 2:**
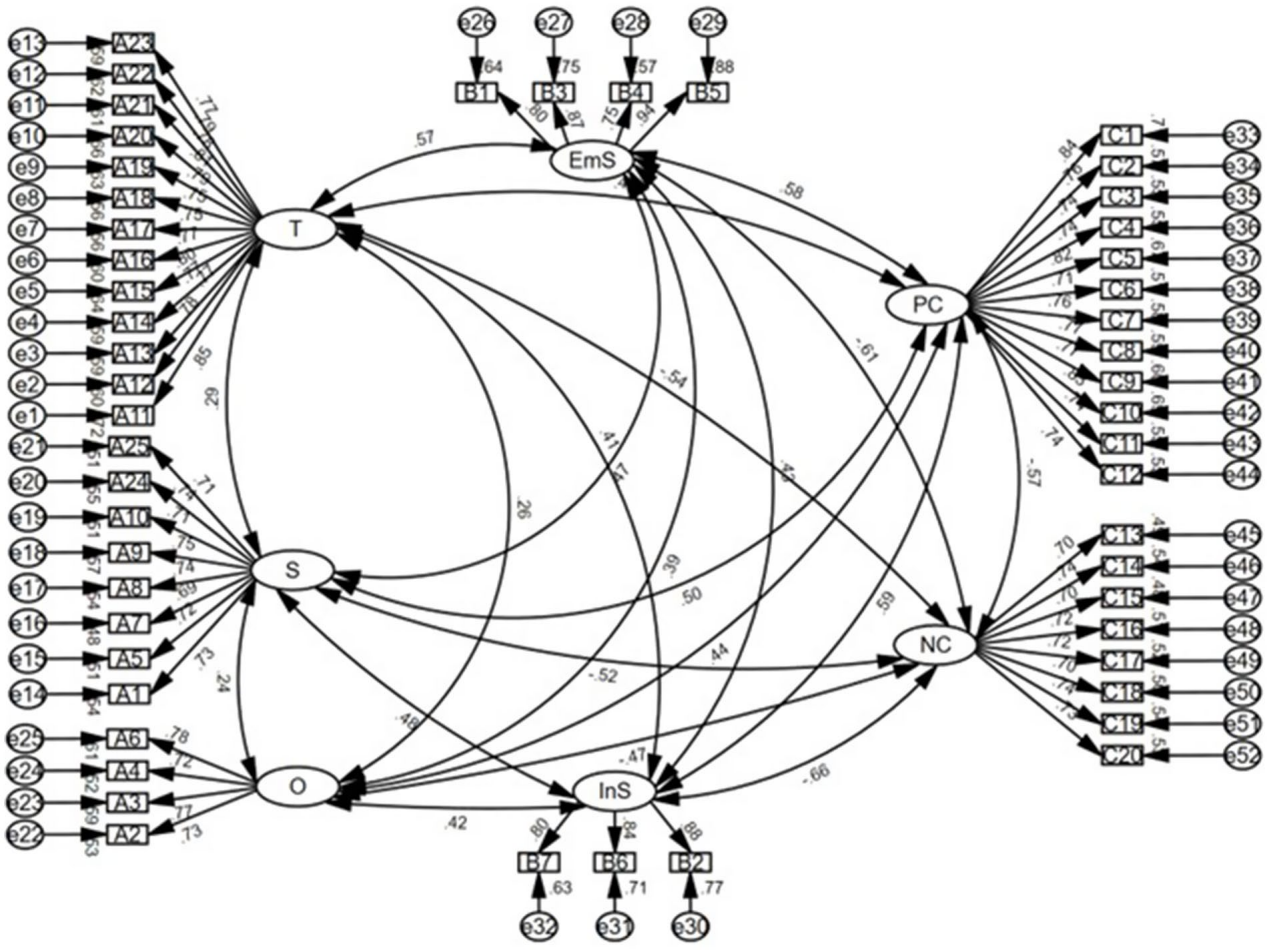
Confirmatory factor analysis of latent variables. T, Tenacity; S, Strength; O, Optimism; EmS, Emotional Support; InS, Instrumental Support; PCB, Positive Coping Behavior; NCB, Negative Coping Behavior.

CFA was conducted to assess the measurement model of PR, SS, and ECB. Figure 2 illustrates the structural model of the latent variables and their measurement items, showing the relationships tested in the CFA. The overall model fit was acceptable. Specifically, the χ2/df was 1.26, which was below the recommended threshold of 3. In addition, RMSEA was 0.03, RMR was 0.04, SRMR was 0.03, and CFI was 0.98, all indicating a good fit of the measurement model. Although GFI (0.88) and AGFI (0.87) did not fully reach the conventional reference value of 0.90, fit evaluation was based on a holistic assessment of multiple indices rather than a single cutoff criterion; therefore, given the satisfactory values of χ2/df , RMSEA, CFI, and SRMR, the measurement model was still considered to demonstrate acceptable overall fit.

Convergent validity was assessed using standardized factor loadings (FL) and average variance extracted (AVE). The standardized factor loadings of all measurement items ranged from 0.70 to 0.94, exceeding the recommended threshold of 0.50, which indicates that the observed items adequately represented their corresponding latent constructs. As shown in Table 4, the square roots of AVE ranged from 0.72 to 0.84, corresponding to AVE values ranging from 0.51 to 0.71, all above the recommended threshold of 0.50. These results provide evidence of acceptable convergent validity.

**Table 4:** Results of construct validity test.

Variables	CR	AVE	T	S	O	EmS	InS	PCB	NCB
T	0.95	0.61	0.78						
S	0.90	0.53	0.27	0.73					
O	0.84	0.56	0.24	0.21	0.75				
EmS	0.91	0.71	0.54	0.43	0.35	0.84			
InS	0.88	0.71	0.38	0.43	0.35	0.39	0.84		
PCB	0.95	0.59	0.47	0.46	0.38	0.55	0.53	0.77	
NCB	0.89	0.51	−0.50	−0.47	−0.41	−0.57	−0.58	−0.52	0.72

Diagonal values are square roots of AVE; off-diagonal values are inter-construct correlations, T, Tenacity; S, Strength; O, Optimism; EmS, Emotional Support; InS, Instrumental Support; PCB, Positive Coping Behavior; NCB, Negative Coping Behavior.

Discriminant validity was examined by comparing the square roots of AVE for each construct with the inter-construct correlations. As shown in Table 4, the square root of the AVE for each construct was greater than its correlations with the other constructs. For example, the square root of the AVE for emotional support was 0.85, which exceeded its correlations with tenacity (0.54), strength (0.43), optimism (0.35), instrumental support (0.40), positive coping behavior (0.55), and negative coping behavior (−0.57). Similar patterns were observed for all other constructs, indicating satisfactory discriminant validity. Overall, the CFA results support the adequacy of the measurement model and confirm that the constructs used in this study demonstrate acceptable convergent and discriminant validity.

### 3.3 Hypothesized model test

SEM was applied using AMOS 31.0 to test the hypothesized relationships among PR, SS, and ECB among university freshmen. The final sample size of 408 was adequate for SEM estimation. In the hypothesized model, tenacity, strength, and optimism were specified as exogenous latent variables; emotional support and instrumental support were specified as mediating variables; and positive coping behavior and negative coping behavior were specified as endogenous variables. The structural model was then used to test all hypotheses.

#### 3.3.1 Structural path testing

**Figure 3:**
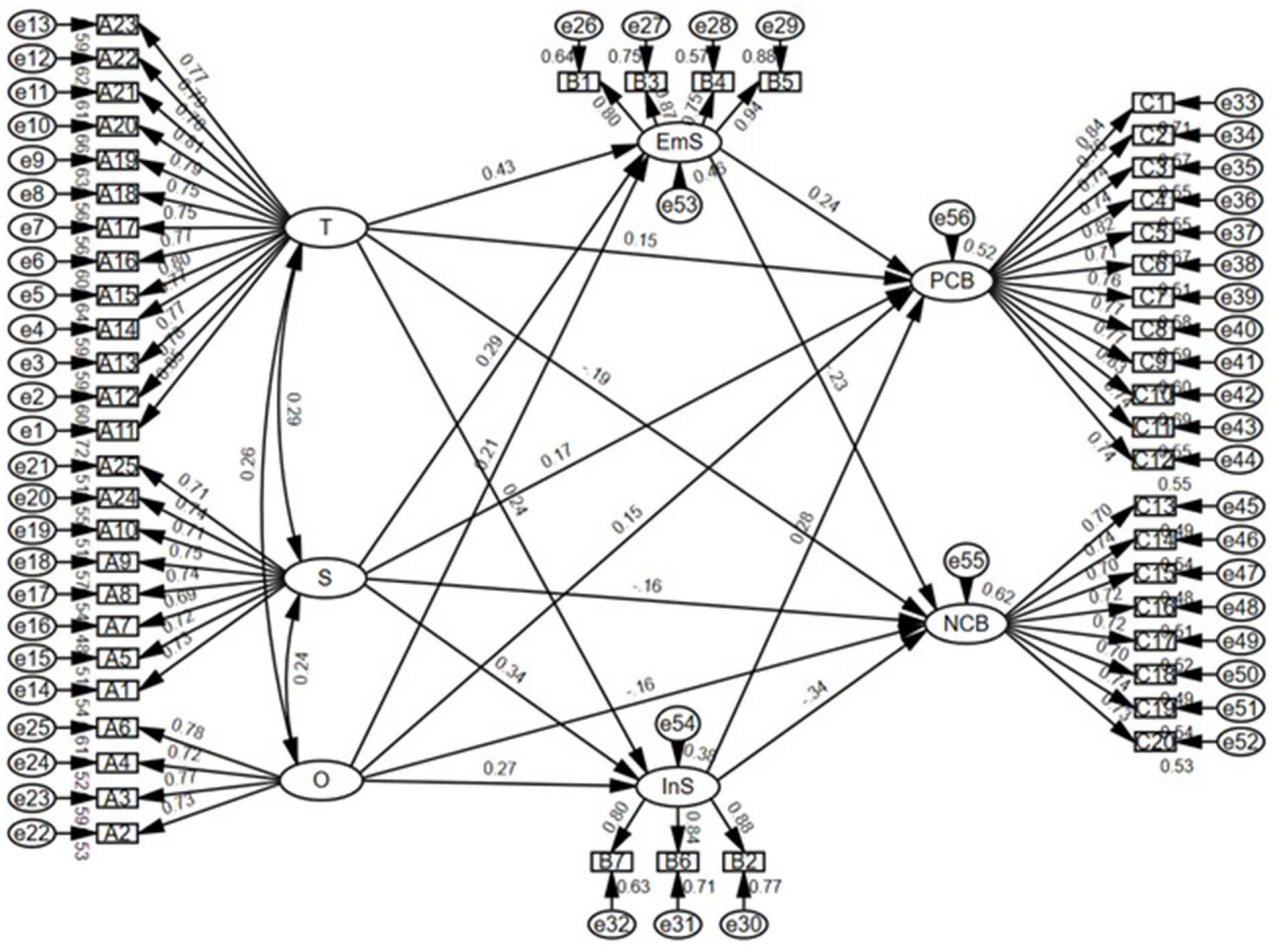
Structural equation model of the hypothesized relationships. T, Tenacity; S, Strength; O, Optimism; EmS, Emotional Support; InS, Instrumental Support; PCB, Positive Coping Behavior; NCB, Negative Coping Behavior.

The hypothesized structural model is shown in Figure 3. The results indicated that the overall structural model demonstrated an acceptable fit to the data ( χ2/df=1.26,RMSEA=0.03,GFI=0.88,CFI=0.98 ), suggesting that the model was appropriate for hypothesis testing.

The results of the structural paths are presented in Table 5. Regarding Hypothesis H1, all three dimensions of PR were significantly associated with ECB in the expected directions: tenacity, strength, and optimism were positively associated with positive coping behavior and negatively associated with negative coping behavior, thus supporting H1a-H1c.

**Table 5:** Results of structural path analysis for the hypothesized model.

Path	Standardized regression weights	S. E.	C. R.	*p*
T→EmS	0.280	0.031	8.905	***
S→EmS	0.255	0.042	6.140	***
O→EmS	0.162	0.038	4.314	***
T→InS	0.213	0.044	4.867	***
S→InS	0.406	0.062	6.532	***
O→InS	0.291	0.057	5.126	***
EmS→PCB	0.336	0.077	4.347	***
InS→PCB	0.285	0.055	5.215	***
EmS→NCB	−0.204	0.050	−4.088	***
InS→NCB	−0.225	0.036	−6.183	***
T→PCB	0.131	0.045	2.939	**
S→PCB	0.206	0.060	3.416	***
O→PCB	0.160	0.053	3.029	**
T→NCB	−0.110	0.029	−3.818	***
S→NCB	−0.129	0.039	−3.339	***
O→NCB	−0.112	0.034	−3.303	***

T, Tenacity; S, Strength; O, Optimism; EmS, Emotional Support; InS, Instrumental Support; PCB, Positive Coping Behavior; NCB, Negative Coping Behavior. ***p < 0.001, **p < 0.01.

Regarding Hypothesis H2, PR was significantly positively associated with SS. Tenacity was positively associated with both emotional support and instrumental support, and strength showed a stronger association with instrumental support than with emotional support, supporting H2a and H2b. Although optimism was positively associated with both forms of support, its association with instrumental support was stronger than with emotional support; therefore, H2c was not supported.

Regarding Hypothesis H3, both emotional support and instrumental support were significantly associated with more positive coping behavior and less negative coping behavior, supporting H3a and H3b. Therefore, the structural path results supported all hypotheses except H2c.

Overall, the structural path results provided empirical support for most of the hypothesized relationships among PR, SS, and ECB among university freshmen.

#### 3.3.2 Mediating effect testing

To further examine Hypothesis H4, the indirect role of SS was tested using the bias-corrected bootstrap method with 5,000 bootstrap samples. A mediating effect was considered significant when the 95% bias-corrected confidence interval did not include zero. As shown in Table 6, all indirect paths from the three dimensions of PR to both positive coping behavior and negative coping behavior through emotional support and instrumental support were statistically significant, and none of the confidence intervals included zero. These results indicate that both emotional support and instrumental support were consistently involved in the indirect associations between PR and ECB. Accordingly, H4a and H4b were supported.

**Table 6:** Significance analysis of mediating effects.

Path	Standardized effect size	Bias-corrected		*p*	Results
		Lower	Upper		
T→EmS→PCB	0.104	0.059	0.161	***	Supported
T→InS→PCB	0.067	0.036	0.110	***	Supported
T→EmS→NCB	−0.097	−0.150	−0.051	***	Supported
T→InS→NCB	−0.081	−0.128	−0.046	***	Supported
S→EmS→PCB	0.072	0.038	0.116	***	Supported
S→InS→PCB	0.097	0.057	0.148	***	Supported
S→EmS→NCB	−0.066	−0.109	−0.034	***	Supported
S→InS→NCB	−0.117	−0.174	−0.075	***	Supported
O→EmS→PCB	0.050	0.024	0.086	***	Supported
O→InS→PCB	0.076	0.042	0.123	***	Supported
O→EmS→NCB	−0.046	−0.081	−0.022	***	Supported
O→InS→NCB	−0.092	−0.139	−0.053	***	Supported

T, Tenacity; S, Strength; O, Optimism; EmS, Emotional Support; InS, Instrumental Support; PCB, Positive Coping Behavior; NCB, Negative Coping Behavior. ***p < 0.001, **p < 0.01.

Overall, the bootstrap results were consistent with SS, represented by emotional support and instrumental support, functioning as an important indirect pathway linking the three dimensions of PR to both positive coping behavior and negative coping behavior among university freshmen. Therefore, Hypothesis H4 was supported. Based on the above hypothesis test results, as shown in Table 7, all hypotheses except H2c were supported.

**Table 7:** Validation results of research hypotheses.

No.	Research hypotheses	Inspection status
H1a	Tenacity is positively associated with positive coping behavior and negatively associated with negative coping behavior.	Supported
H1b	Strength is positively associated with positive coping behavior and negatively associated with negative coping behavior.	Supported
H1c	Optimism is positively associated with positive coping behavior and negatively associated with negative coping behavior.	Supported
H2a	Tenacity is positively associated with both emotional support and instrumental support.	Supported
H2b	Strength is positively associated with both emotional support and instrumental support, with a stronger association with instrumental support.	Supported
H2c	Optimism is positively associated with both emotional support and instrumental support, with a stronger association with emotional support.	Unsupported
H3a	Emotional support is positively associated with positive coping behavior and negatively associated with negative coping behavior.	Supported
H3b	Instrumental support is positively associated with positive coping behavior and negatively associated with negative coping behavior.	Supported
H4a	Emotional support mediates the relationship between the dimensions of psychological resilience and both positive coping behavior and negative coping behavior.	Supported
H4b	Instrumental support mediates the relationship between the dimensions of psychological resilience and both positive coping behavior and negative coping behavior.	Supported

## 4 Discussion

This study examined the associations among PR, SS, and ECB among university freshmen. Overall, the results were broadly consistent with the proposed model. Higher levels of tenacity, strength, and optimism were associated with more positive coping behavior and less negative coping behavior; higher levels of PR were also associated with greater emotional support and instrumental support; and both forms of support were, in turn, associated with ECB in the expected directions. In addition, the SEM results were consistent with SS functioning as an indirect pathway linking PR and ECB. These findings provide a useful basis for understanding how internal psychological resources and external support resources may be jointly related to freshmen’s emergency coping during the transition to university life.

First, the observed associations between PR and ECB are generally consistent with prior research showing that resilience is linked to more adaptive coping tendencies among university students (21). On this basis, the present study focused specifically on university freshmen, a population undergoing a critical transitional stage characterized by adaptation to unfamiliar environments, reconstruction of social relationships, and still-developing supportive networks. By examining in greater detail the associations between different dimensions of PR and different forms of ECB, this study extends existing research beyond broad general discussions of resilience and adaptation. The results suggest that freshmen reporting stronger persistence, greater self-confidence, and more positive expectations also tended to report more adaptive coping tendencies in emergency-related contexts. The results provide a more fine-grained understanding of how internal psychological resources are linked to behavioral responses under stress and further enrich the current literature on university students’ emergency coping.

Second, in the present study, freshmen with higher levels of PR tended to report greater emotional support and instrumental support, suggesting that internal psychological resources may be related not only to how students cope on their own, but also to how they perceive, seek, and use interpersonal resources in unfamiliar environments. The positive associations between PR and SS are also compatible with earlier findings on student adaptation and support processes (8, 43). At the same time, one result deserves particular attention: optimism was more strongly associated with instrumental support than with emotional support, contrary to the original expectation. Compared with studies emphasizing optimism’s affective and relational benefits (35), the present result suggests that in emergency-related contexts, optimistic freshmen may orient more readily toward practical guidance, useful information, and actionable assistance than toward affective reassurance alone. This pattern may reflect the specific demands of the freshman transition, in which concrete help and navigational support are especially salient.

Third, the observed associations between SS and ECB reinforce the view that support resources are not merely contextual background variables but are closely related to coping tendencies. The present results showed that both emotional support and instrumental support were associated with more positive coping behavior and less negative coping behavior. This is broadly consistent with prior studies showing that greater perceived SS is related to more active coping and lower distress among college students (28, 34). The present findings add to this literature by distinguishing between emotional support and instrumental support within one model. The results suggest that emotional support may be more closely linked to psychological stabilization under stress, whereas instrumental support may be more closely linked to situational understanding and organized action. This distinction is meaningful in the freshman context, because students entering university may require not only reassurance and belonging, but also timely information, practical guidance, and identifiable help channels when emergencies occur.

Most importantly, the indirect pattern observed in the SEM analysis suggests that SS may help explain how PR and ECB are connected. Earlier work has already hinted at such a mechanism (20, 41). The present study contributes to this broader literature by examining this indirect pattern specifically among university freshmen and by distinguishing both forms of SS and both forms of ECB. In this sense, the study adds a more fine-grained account of the interrelationships among resilience, support, and coping in a transition-stage student population.

The findings also have practical implications. Because emotional support and instrumental support were both associated with ECB, campus responses should not be limited to general appeals for “resilience training.” Instead, universities may use these findings to identify concrete support targets during the freshman transition. For example, the association between emotional support and coping suggests the value of making reassurance and interpersonal support more visible during emergency-related adjustment, such as through peer mentoring, counselor accessibility, resident advisor check-ins, and structured first-year support groups. Similarly, the association between instrumental support and coping suggests the importance of ensuring that freshmen can easily access authoritative information, clear emergency procedures, referral pathways, and practical help channels. In particular, because strength and optimism were more strongly associated with instrumental support in the present sample, universities may consider whether some freshmen need not only emotional encouragement but also clearer action guidance and more accessible support infrastructure when facing uncertainty.

Several limitations should be acknowledged. First, the cross-sectional design does not permit strong causal inference. Although the SEM results were consistent with the hypothesized model, longitudinal or experimental research is needed to determine temporal order and causal direction more rigorously. Second, the study relied entirely on self-report questionnaires, which may have introduced common method bias, recall bias, and social desirability effects. Third, the sample was drawn from university freshmen in a specific institutional and cultural context, which may limit generalizability to students at other stages, to other universities, or to non-Chinese populations. Fourth, several potentially relevant confounding factors were not explicitly controlled in the present study, including prior emergency experience, baseline mental health status, personality characteristics, family socioeconomic background, and pre-existing levels of SS. Finally, although the constructs were theoretically distinguished and the measurement model showed acceptable discriminant validity, some conceptual overlap among the instruments cannot be fully ruled out. Although support utilization was not modeled as a separate dimension of SS in the present study in order to reduce this problem, such overlap may still have contributed to some of the observed correlations. Future studies could address these issues by using multi-site samples, longitudinal designs, event-specific coping measures, multi-informant or behavioral indicators, and sensitivity analyses.

Overall, the present study suggests that PR, SS, and ECB are meaningfully interconnected among university freshmen. By examining PR and SS as multidimensional constructs and by distinguishing positive coping behavior from negative coping behavior, this study contributes a more differentiated account of the correlational pattern linking internal resources, external support, and emergency coping tendencies during the transition to university life.

## 5 Conclusion

Using questionnaire data from 408 university freshmen, this study employed SEM to examine the relationships among PR, SS, and ECB. The main conclusions are as follows:

1. PR was significantly associated with ECB among university freshmen. Higher levels of tenacity, strength, and optimism were associated with more positive coping behavior and less negative coping behavior, indicating that PR is an important internal resource for adaptive emergency response.
2. PR was significantly positively associated with SS. Freshmen with higher levels of resilience reported higher levels of both emotional support and instrumental support, suggesting that resilience is positively related to the development and mobilization of interpersonal support resources during the transition to university life. Furthermore, both strength and optimism had a stronger influence on instrumental support. This may be because instrumental support is more urgently needed than emotional support during the early stage of university adaptation.
3. SS was significantly associated with ECB. Both emotional support and instrumental support were associated with more positive coping behavior and less negative coping behavior, indicating that external support resources are closely related to freshmen’s emergency responses.
4. The SEM results were consistent with SS functioning as a significant indirect pathway in the relationship between PR and ECB. This finding suggests that PR may be linked to coping not only directly, but also indirectly through greater emotional and instrumental support.

Overall, this study suggests that ECB among university freshmen is meaningfully associated with both PR and SS. The findings suggest that interventions aimed at improving freshmen’s emergency coping should address both internal psychological resources and external support resources. Specifically, universities may strengthen freshmen’ resilience while also ensuring that emotional reassurance, practical assistance, and clear help-seeking channels are accessible during the transition to university life. By clarifying both the direct and indirect pathways through which PR influences ECB, this study deepens current understanding of freshmen’s adaptation in emergency contexts and offers useful implications for higher education management. At the same time, the unsupported expectation regarding optimism suggests that the effects of specific resilience dimensions on different forms of support may vary across contexts and deserve further investigation.

## Data Availability

The original contributions presented in the study are included in the article/Supplementary material, further inquiries can be directed to the corresponding author.
