# Supplementary material for: Psychological resilience and emergency coping behavior among university freshmen: the mediating role of social support
Source: Front Public Health. 2026 May 13;14:1831841. doi: 10.3389/fpubh.2026.1831841 (PMC13212512; doi:10.3389/fpubh.2026.1831841)
Supplement: Supplementary file 2 [file Table_2.DOCX]

**Survey on Psychological Resilience, Social Support, and Emergency Coping Behavior among University Freshmen**

# Section 1. Demographic Information

Please complete the following information by ticking the appropriate option.

| **Item** | **Options** | | | | | |
| --- | --- | --- | --- | --- | --- | --- |
| **Gender** | □Male | | | □Female | | |
| **Age** | □17 years | □18 years | | | □19 years and above | |
| **Place of origin** | □ Urban | | | □ Rural | | |
| **Only-child status** | □ Yes | | | □ No | | |
| **Academic discipline** | □ Science | | □ Engineering | | | □ Medicine |
|  | □ Economics | | □ Law | | | □ Literature |
|  | □ Philosophy | | □ Arts | | | □ Others |

# Section II. Psychological Resilience Scale (CD-RISC)

**Instructions:**

Dear participant,

This questionnaire is a self-report scale used to assess psychological resilience. It is designed to measure your ability to adapt and cope when facing stress and adversity. The questionnaire contains 25 items. There are no right or wrong answers. Please respond to each statement according to your actual situation over the past month. Please do not spend too much time thinking about each item; simply choose the answer that best reflects your first impression. All responses will be used for academic research only, and your personal information will be kept strictly confidential.

**Response instructions:**

Please tick (√) one option for each item. Each item should have only one answer.

**Response options:**

0 = Never 1 = Rarely 2 = Sometimes 3 = Often 4 = Always

**Questionnaire items:**

| **No.** | **Item** | **0**  **Never** | **1 Rarely** | **2 Sometimes** | **3 Often** | **4 Always** |
| --- | --- | --- | --- | --- | --- | --- |
| 1 | I am able to adapt to change. | □ | □ | □ | □ | □ |
| 2 | I have close and secure relationships. | □ | □ | □ | □ | □ |
| 3 | Sometimes fate or God can help. | □ | □ | □ | □ | □ |
| 4 | I can deal with whatever comes. | □ | □ | □ | □ | □ |
| 5 | Past successes give me confidence in dealing with new challenges. | □ | □ | □ | □ | □ |
| 6 | I can see the humorous side of things. | □ | □ | □ | □ | □ |
| 7 | Coping with stress can make me stronger. | □ | □ | □ | □ | □ |
| 8 | I tend to recover quickly after hardship or illness. | □ | □ | □ | □ | □ |
| 9 | Things happen for a reason. | □ | □ | □ | □ | □ |
| 10 | I give my best effort no matter what the outcome may be. | □ | □ | □ | □ | □ |
| 11 | I can achieve my goals. | □ | □ | □ | □ | □ |
| 12 | When things look hopeless, I do not give up easily. | □ | □ | □ | □ | □ |
| 13 | I know where to turn for help. | □ | □ | □ | □ | □ |
| 14 | Under pressure, I can stay focused and think clearly. | □ | □ | □ | □ | □ |
| 15 | I like to take the lead in solving problems. | □ | □ | □ | □ | □ |
| 16 | I am not easily discouraged by failure. | □ | □ | □ | □ | □ |
| 17 | I think of myself as a strong person. | □ | □ | □ | □ | □ |
| 18 | I can make unusual or difficult decisions. | □ | □ | □ | □ | □ |
| 19 | I can handle unpleasant feelings such as sadness and anger. | □ | □ | □ | □ | □ |
| 20 | I sometimes have to act on intuition. | □ | □ | □ | □ | □ |
| 21 | I have a strong sense of purpose. | □ | □ | □ | □ | □ |
| 22 | I feel in control of my life. | □ | □ | □ | □ | □ |
| 23 | I like challenges. | □ | □ | □ | □ | □ |
| 24 | I work hard to attain my goals. | □ | □ | □ | □ | □ |
| 25 | I am proud of my achievements. | □ | □ | □ | □ | □ |

# Section III. Social Support Rating Scale (SSRS)

**Instructions:**

This questionnaire is used to assess the level of social support you receive in your daily life. All responses are for academic research only and will be kept strictly confidential. The questionnaire is anonymous.

Please answer each question according to your actual situation. There are no right or wrong answers. Please complete the questionnaire independently and do not discuss your answers with others. Thank you for your cooperation.

**Questionnaire Items:**

**1. How many close friends do you have who can provide you with support and help?**

□ (1) None □ (2) 1–2 □ (3) 3–5 □ (4) 6 or more

**2. In the past year, your living situation has mainly been:**

□ (1) Living alone and away from family

□ (2) Living in a collective dormitory

□ (3) Living with friends or colleagues

□ (4) Living with family members

**3. Your relationship with your neighbors is:**

□ (1) No contact at all

□ (2) Occasional contact

□ (3) Frequent contact

□ (4) Very close contact

**4. Your relationship with your classmates or colleagues is:**

□ (1) No contact at all

□ (2) Occasional contact

□ (3) Frequent contact

□ (4) Very close contact

**5. The support and care you receive from family members**

(Please tick one option for each person.)

| **Family member** | **None** | **Very little** | **Moderate** | **Full support** |
| --- | --- | --- | --- | --- |
| a. Boyfriend/Girlfriend or Spouse | □ | □ | □ | □ |
| b. Parents | □ | □ | □ | □ |
| c. Siblings | □ | □ | □ | □ |
| d. Other family members | □ | □ | □ | □ |

**6. In times of emergency or difficulty, what sources have provided you with financial support or practical help?**

(Multiple choices allowed)

□ (1) No source

□ (2) Boyfriend/Girlfriend or Spouse

□ (3) Parents

□ (4) Siblings

□ (5) Friends

□ (6) Classmates or Colleagues

□ (7) School or Work Unit

□ (8) Party/League, Trade Union, or Social Organizations

□ (9) Other

**7. In times of emergency or difficulty, what sources have provided you with comfort and care?**

(Multiple choices allowed)

□ (1) No source

□ (2) Boyfriend/Girlfriend or Spouse

□ (3) Parents

□ (4) Siblings

□ (5) Friends

□ (6) Classmates or Colleagues

□ (7) School or Work Unit

□ (8) Party/League, Trade Union, or Social Organizations

□ (9) Other

**8. When you feel troubled, how do you usually talk about it?**

□ (1) I never talk to anyone

□ (2) I only talk to one or two people with whom I am extremely close

□ (3) I talk about it only if a friend asks me

□ (4) I actively talk about it in order to gain support and understanding

**9. When you feel troubled, how do you usually seek help?**

□ (1) I rely only on myself and do not accept help from others

□ (2) I rarely ask others for help

□ (3) I sometimes ask others for help

□ (4) I actively seek help from family members, friends, or organizations

**10. Participation in group or organizational activities:**

□ (1) Never participate

□ (2) Occasionally participate

□ (3) Frequently participate

□ (4) Actively participate and help organize activities

# Section IV. Simplified Coping Style Questionnaire (SCSQ)

**Instructions:**

This questionnaire is a self-report scale used to assess the coping styles you usually adopt when facing stress or difficult situations. It aims to understand how you typically respond when you encounter difficulties or stressful events. The questionnaire contains 20 items. There are no right or wrong answers. Please choose the option that best reflects your actual situation. Please do not overthink your answers; respond according to your first impression. All responses will be used for academic research only and will be kept strictly confidential.

**Response options:**

1 = Never use 2 = Occasionally use 3 = Sometimes use 4 = Often use

Please tick (√) one option for each item.

**Questionnaire Items:**

| **No.** | **Item** | **1**  **Never use** | **2 Occasionally use** | **3 Sometimes use** | **4**  **Often use** |
| --- | --- | --- | --- | --- | --- |
| 1 | I try to get rid of my troubles through work or study. | □ | □ | □ | □ |
| 2 | I talk with others and express my inner distress. | □ | □ | □ | □ |
| 3 | I try to see the positive side of things. | □ | □ | □ | □ |
| 4 | I change my way of thinking and rediscover the meaning of life. | □ | □ | □ | □ |
| 5 | I do not take the problem too seriously. | □ | □ | □ | □ |
| 6 | I stick to my position and strive for myself. | □ | □ | □ | □ |
| 7 | I try to find several different ways to solve the problem. | □ | □ | □ | □ |
| 8 | I seek advice from relatives, friends, or classmates. | □ | □ | □ | □ |
| 9 | I change some of my original practices or my own problems. | □ | □ | □ | □ |
| 10 | I learn from how others deal with similar difficult situations. | □ | □ | □ | □ |
| 11 | I develop hobbies and actively participate in cultural or sports activities. | □ | □ | □ | □ |
| 12 | I try to restrain my feelings of disappointment, regret, sadness, and anger. | □ | □ | □ | □ |
| 13 | I try to rest or take a vacation and temporarily put the problem aside. | □ | □ | □ | □ |
| 14 | I relieve my troubles by smoking, drinking, taking medicine, or eating. | □ | □ | □ | □ |
| 15 | I believe that time will change the situation, and the only thing to do is wait. | □ | □ | □ | □ |
| 16 | I try to forget the whole thing. | □ | □ | □ | □ |
| 17 | I rely on others to solve the problem. | □ | □ | □ | □ |
| 18 | I accept reality because there is no other way. | □ | □ | □ | □ |
| 19 | I imagine that some miracle may happen to change the current situation. | □ | □ | □ | □ |
| 20 | I comfort myself. | □ | □ | □ | □ |
